# High oxide-ion conductivity through the interstitial oxygen site in Ba_7_Nb_4_MoO_20_-based hexagonal perovskite related oxides

**DOI:** 10.1038/s41467-020-20859-w

**Published:** 2021-01-25

**Authors:** Masatomo Yashima, Takafumi Tsujiguchi, Yuichi Sakuda, Yuta Yasui, Yu Zhou, Kotaro Fujii, Shuki Torii, Takashi Kamiyama, Stephen J. Skinner

**Affiliations:** 1 Department of Chemistry, School of Science, Tokyo Institute of Technology, 2–12–1 W4–17 O-okayama, Meguro-ku, Tokyo, 152–8551 Japan; 2 Department of Materials, Imperial College London, Exhibition Road, London, SW7 2AZ UK; 3 Institute of Materials Structure Science, High Energy Accelerator Research Organization (KEK), 203-1 Tokai, Ibaraki, 319–1106 Japan

**Keywords:** Energy, Fuel cells, Fuel cells

## Abstract

Oxide-ion conductors are important in various applications such as solid-oxide fuel cells. Although zirconia-based materials are widely utilized, there remains a strong motivation to discover electrolyte materials with higher conductivity that lowers the working temperature of fuel cells, reducing cost. Oxide-ion conductors with hexagonal perovskite related structures are rare. Herein, we report oxide-ion conductors based on a hexagonal perovskite-related oxide Ba_7_Nb_4_MoO_20_. Ba_7_Nb_3.9_Mo_1.1_O_20.05_ shows a wide stability range and predominantly oxide-ion conduction in an oxygen partial pressure range from 2 × 10^−26^ to 1 atm at 600 °C. Surprisingly, bulk conductivity of Ba_7_Nb_3.9_Mo_1.1_O_20.05_, 5.8 × 10^−4^ S cm^−1^, is remarkably high at 310 °C, and higher than Bi_2_O_3_- and zirconia-based materials. The high conductivity of Ba_7_Nb_3.9_Mo_1.1_O_20.05_ is attributable to the interstitial-O5 oxygen site, providing two-dimensional oxide-ion O1−O5 interstitialcy diffusion through lattice-O1 and interstitial-O5 sites in the oxygen-deficient layer, and low activation energy for oxide-ion conductivity. Present findings demonstrate the ability of hexagonal perovskite related oxides as superior oxide-ion conductors.

## Abstract (web summary)

Oxide-ion conductors are important in various applications for clean energy. Here, authors report high oxide-ion conductivity of hexagonal perovskite-related oxide Ba_7_Nb_3.9_Mo_1.1_O_20.05_, which is ascribed to the interstitialcy diffusion and low activation energy for oxide-ion conductivity.

## Introduction

Oxide-ion conducting ceramic materials have attracted much attention due to their applications in solid-oxide fuel cells (SOFCs), oxygen separation membranes, gas sensors, and catalysts^1–24^. Yttria stabilized zirconia (YSZ) ceramics have widely been used, but the working temperature is high (700–1000 °C). Thus, there remains a strong motivation to explore oxide-ion conductors with higher conductivities at temperatures below 600 °C. High oxide-ion conductivities have been observed in specific structure families such as the fluorite-type, perovskite-type, melilite-type, and apatite-type structures^1–24^.

The perovskite and its related materials exhibit interesting physical and chemical properties^25^ and can be classified into four structural groups of (i) *AMX*_3_ perovskite-type, (ii) *AMX*_3_-related, (iii) hexagonal perovskite-related and (iv) modular structures^26^ where *A* and *M* are larger and smaller cations, respectively, and *X* is an anion. A number of perovskite-type materials and perovskite related phases belonging to the groups of (i), (ii) and (iv) have been reported to be oxide-ion conductors. The hexagonal perovskite-related structures (iii) have hexagonal close packing of *AX*_3_ layers or sequences of hexagonal (h) and cubic (c) *AX*_3_ (and/or anion-deficient *AX*_3–*x*_ (c′) where *x* is the anion vacancy content) close-packed layers. The hexagonal perovskite related oxides exhibit a variety of crystal structures^26–31^. However, oxide-ion conductors with hexagonal perovskite-related structures are quite rare. Several mixed ion (oxide-ion and/or proton) and electronic conductors with hexagonal perovskite related structures were reported in the literature^32–35^. The hexagonal perovskite related oxides Ba_3_*M*NbO_8.5–*δ*_ (*M* = Mo and W; *δ* is the oxygen deficiency) and their solid solutions exhibit significant oxide-ion conductivities^23,30,31,36–39^, however, the conductivities are not high at temperatures below 600 °C. The relatively low conductivities are ascribed to relatively high activation energy for conductivity (e.g., 1.2 eV for Ba_3_MoNbO_8.5–*δ*_^23^). Therefore, we have explored oxide-ion conductors with the hexagonal perovskite related structures. Ba_7_Nb_4_MoO_20_ is a trigonal $P\bar 3m1$ hexagonal perovskite polytype 7H^29,40^. Fop et al. found high oxide-ion and proton conductivities of Ba_7_Nb_4_MoO_20_^40^. Herein, we report higher oxide-ion conductivities, crystal structure and oxide-ion diffusion pathways of the solid solution composition Ba_7_Nb_3.9_Mo_1.1_O_20.05_. Ba_7_Nb_3.9_Mo_1.1_O_20.05_ is found to exhibit a bulk conductivity of 5.8 × 10^−4^ S cm^−1^ at 310 °C, which is higher than those of the “best” oxide-ion conductors. The present work also demonstrates the two-dimensional (2D) oxide-ion O1–O5 diffusion through the interstitial octahedral O5 and lattice tetrahedral O1 sites at a high temperature of 800 °C.

## Results and discussion

### Preparation and characterization of Ba_7_Nb_4_MoO_20_-based oxides

In this work, we studied the electrical and structural properties of Ba_7_Nb_4_MoO_20_-based materials, because (1) the chemical species in Ba_7_Nb_4_MoO_20_ are the same as those in the oxide-ion conductor Ba_3_MoNbO_8.5–*δ*_, (2) Ba_7_Nb_4_MoO_20_ has the hexagonal perovskite related structure similar to Ba_3_MoNbO_8.5–*δ*_^28,29,38^, (3) the crystal structure of Ba_7_Nb_4_MoO_20_ contains possible oxide-ion conducting Ba–oxygen (c′) layers as does the structure of Ba_3_MoNbO_8.5-*δ*_, and (4) the bond-valence-based energy barrier for oxide-ion migration of Ba_7_Nb_4_MoO_20_ (0.21 eV) is lower than that of Ba_3_MoNbO_8.5–*δ*_ (0.51–0.35 eV, See the details in Supplementary Note 1). Ba_7_Nb_3.95_Mo_1.05_O_20.025_ and Ba_7_Nb_3.9_Mo_1.1_O_20.05_ were successfully prepared by solid-state reactions. X-ray powder diffraction (XRD) measurements indicated that Ba_7_Nb_3.95_Mo_1.05_O_20.025_ and Ba_7_Nb_3.9_Mo_1.1_O_20.05_ have the hexagonal perovskite related structure with trigonal $P\bar 3m1$ space group (Supplementary Fig. 1). Arrhenius plots of bulk conductivities (*σ*_b_) of Ba_7_Nb_4_MoO_20_^40^, Ba_7_Nb_3.95_Mo_1.05_O_20.025_, and Ba_7_Nb_3.9_Mo_1.1_O_20.05_ in dry air are shown in Supplementary Fig. 2. The *σ*_b_ of Ba_7_Nb_3.9_Mo_1.1_O_20.05_ is the highest among the three compositions. Thus, we focus on the Ba_7_Nb_3.9_Mo_1.1_O_20.05_ composition for further detailed studies.

The cation atomic ratio of Ba: Nb: Mo = 7.11(14): 3.81(3): 1.126(14) for Ba_7_Nb_3.9_Mo_1.1_O_20.05_ determined through X-ray fluorescence (XRF) analyses agreed with that of the nominal composition where the number in parentheses is the standard deviation in the last digit. X-ray photoelectron spectroscopy (XPS) data for the Ba_7_Nb_3.9_Mo_1.1_O_20.05_ composition indicated that the valences of Ba, Nb and Mo at room temperature (RT) were +2, +5 and +6, respectively (Ba^2+^_7_Nb^5+^_3.9_Mo^6+^_1.1_O^2–^_20.05_; Supplementary Fig. 3). Thermogravimetric measurements of Ba_7_Nb_3.9_Mo_1.1_O_20.05_ in dry air between 400 and 900 °C indicate very little weight loss and oxygen deficiency *δ* in Ba_7_Nb_3.9_Mo_1.1_O_20.05−*δ*_ at high temperatures (Supplementary Fig. 4).

### Oxide-ion conduction in Ba_7_Nb_3.9_Mo_1.1_O_20.05_

**Fig. 1:**
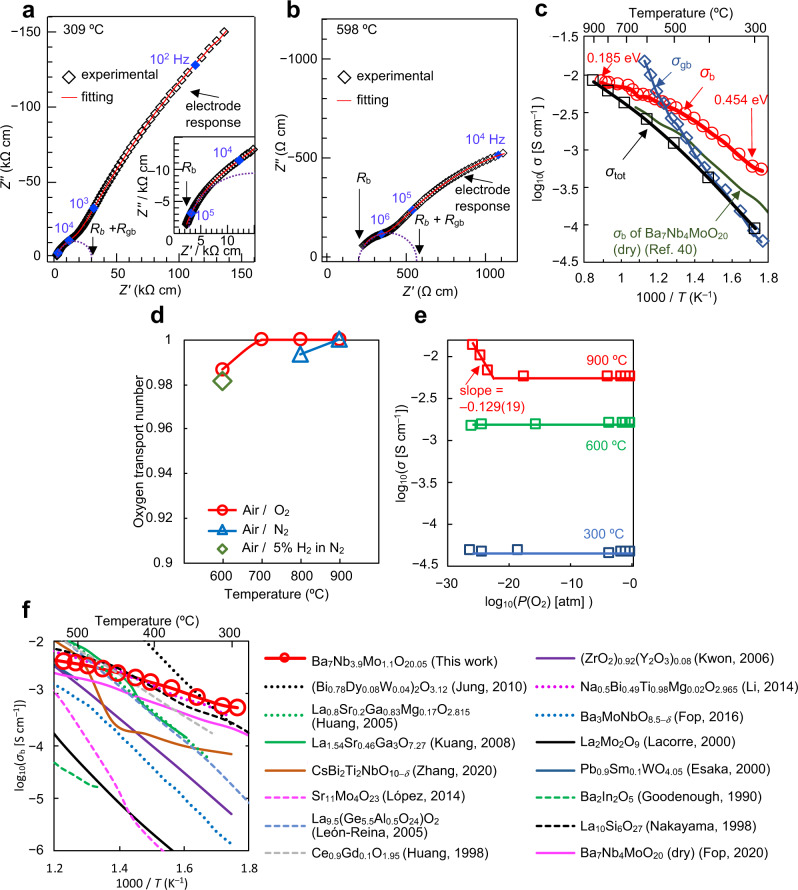
High oxide-ion conductivity of Ba_7_Nb_3.9_Mo_1.1_O_20.05_. **a**, **b** Complex impedance plots of Ba_7_Nb_3.9_Mo_1.1_O_20.05_ recorded in dry air at (**a**) 309 °C and (**b**) 598 °C. **c** Arrhenius plots of bulk conductivity *σ*_b_, grain-boundary conductivity *σ*_gb_ and DC *σ*_tot_ of Ba_7_Nb_3.9_Mo_1.1_O_20.05_ in dry air. Activation energy for *σ*_b_ of Ba_7_Nb_3.9_Mo_1.1_O_20.05_ decreases with temperature from 0.454 to 0.185 eV as shown by the red numbers in panel c. Green line represents *σ*_b_ of Ba_7_Nb_4_MoO_20_ reported by Fop et al.^40^. **d** Oxygen transport number of Ba_7_Nb_3.9_Mo_1.1_O_20.05_. **e** Oxygen partial pressure *P*(O_2_) dependence of *σ*_tot_ of Ba_7_Nb_3.9_Mo_1.1_O_20.05_. **f** Comparison of bulk conductivities of Ba_7_Nb_3.9_Mo_1.1_O_20.05_ and other oxide-ion conductors.

Figure 1a, b shows the typical impedance spectra of Ba_7_Nb_3.9_Mo_1.1_O_20.05_, which indicates the bulk, grain boundary and electrode responses. Bulk conductivity (*σ*_b_), grain-boundary conductivity (*σ*_gb_), and grain-boundary capacitance were obtained by the equivalent circuit fitting (Red lines in Fig. 1a, b, Supplementary Figures 5–9). The *σ*_b_ and *σ*_gb_ were measured in dry O_2_, dry air and in dry N_2_ at 295 and 598 °C. They were independent of oxygen partial pressure at these temperatures, which indicates ionic conduction (Supplementary Figure 10). Figure 1c shows the temperature dependencies of the *σ*_b_ and *σ*_gb_ of Ba_7_Nb_3.9_Mo_1.1_O_20.05_ on cooling in dry air. The activation energy for *σ*_b_ was found to be lower than those for *σ*_gb_ and the DC total electrical conductivity, *σ*_tot_. The *σ*_b_ was higher than *σ*_gb_ at temperatures below 550 °C and higher than *σ*_tot_ at temperatures below 850 °C. The oxide-ion transport number (*t*_ion_) was investigated using oxygen concentration cell measurements. The *t*_ion_ values were 1.00 between 700 and 900 °C and 0.99 at 600 °C in air/O_2_, 0.99 at 800 °C and 1.00 at 900 °C in air/N_2_, and 0.98 at 600 °C in air/5% H_2_ in N_2_ (Fig. 1d). Oxide-ion diffusion in dense Ba_7_Nb_3.9_Mo_1.1_O_20.05_ was clearly confirmed by the isotope exchange depth profile method^41^, which calculated a high oxygen tracer diffusion coefficient D* value of 5.35 × 10^–9^ cm^2^ s^–1^ at 700 °C and 7.25 × 10^–9^ cm^2^ s^–1^ at 800 °C (Supplementary Figure 11). The diffusion lengths were about 150 μm and the grain sizes of the Ba_7_Nb_3.9_Mo_1.1_O_20.05_ samples were 1–5 μm (Supplementary Figure 12), thus, the ^18^O tracer species encountered a number of grains and grain boundaries. The total DC electrical conductivity (*σ*_tot_) was independent of the oxygen partial pressure *P*(O_2_) between *P*(O_2_) = 7 × 10^−25^ ~ 1 atm at 300 °C, *P*(O_2_) = 2 × 10^−26^ ~ 1 atm at 600 °C, and *P*(O_2_) = 5 × 10^−18^ ~ 1 atm at 900 °C (Fig. 1e). Electronic conductivity was observed in the *P*(O_2_) range of 6 × 10^−24^ ~ 4 × 10^−26^ atm at 900 °C with the slope of −0.129(19). Therefore, Ba_7_Nb_3.9_Mo_1.1_O_20.05_ exhibits a wider electrolyte domain compared with Ba_7_Nb_4_MoO_20_ as reported by Fop et al.^40^. To examine the possible proton conduction of this phase, the DC electrical conductivities, *σ*_tot_, of Ba_7_Nb_3.9_Mo_1.1_O_20.05_ were measured in wet air (H_2_O partial pressure, *P*(H_2_O) = 2.3 × 10^−2^ atm) and in dry air (*P*(H_2_O) < 1.8 × 10^−4^ atm). The contribution of protons to *σ*_tot_ of Ba_7_Nb_3.9_Mo_1.1_O_20.05_ was small, even in wet air where the proton transport number was 0.03 − 0.12 (Supplementary Fig. 13). Water incorporation behavior was also investigated and the results are shown in Supplementary Fig. 14 and Supplementary Note 2. A significant but small amount of water uptake was observed for Ba_7_Nb_3.9_Mo_1.1_O_20.05_ compared with Ba_7_Nb_4_MoO_20_, which is responsible for the lower transport number of protons in Ba_7_Nb_3.9_Mo_1.1_O_20.05_. These results indicate that the oxide ion is the dominant charge carrier and that Ba_7_Nb_3.9_Mo_1.1_O_20.05_ is an oxide-ion conductor. No change was observed in the X-ray powder diffraction patterns before and after the oxygen concentration cell measurements (Supplementary Fig. 15), which demonstrates the high phase stability of Ba_7_Nb_3.9_Mo_1.1_O_20.05_ at high temperatures and in the wide *P*(O_2_) region between *P*(O_2_) = 1.2 × 10^−27^ and 1 atm. The *σ*_b_ of Ba_7_Nb_3.9_Mo_1.1_O_20.05_ was found to be higher than those of Ba_7_Nb_4_MoO_20_^40^ (Fig. 1c) and YSZ, and comparable to those of the best oxide-ion conductors (Fig. 1f). It should be noted that the *σ*_b_ of Ba_7_Nb_3.9_Mo_1.1_O_20.05_ was higher than the best oxide-ion conductors at temperatures of around 300 °C, due to the low activation energy of Ba_7_Nb_3.9_Mo_1.1_O_20.05_ (0.185-0.454 eV as shown in Fig. 1c). These results indicate the high potential of the hexagonal perovskite related oxide Ba_7_Nb_3.9_Mo_1.1_O_20.05_ as a superior oxide-ion conductor.

### Structural origin of the high oxide-ion conductivity in Ba_7_Nb_3.9_Mo_1.1_O_20.05_

**Fig. 2:**
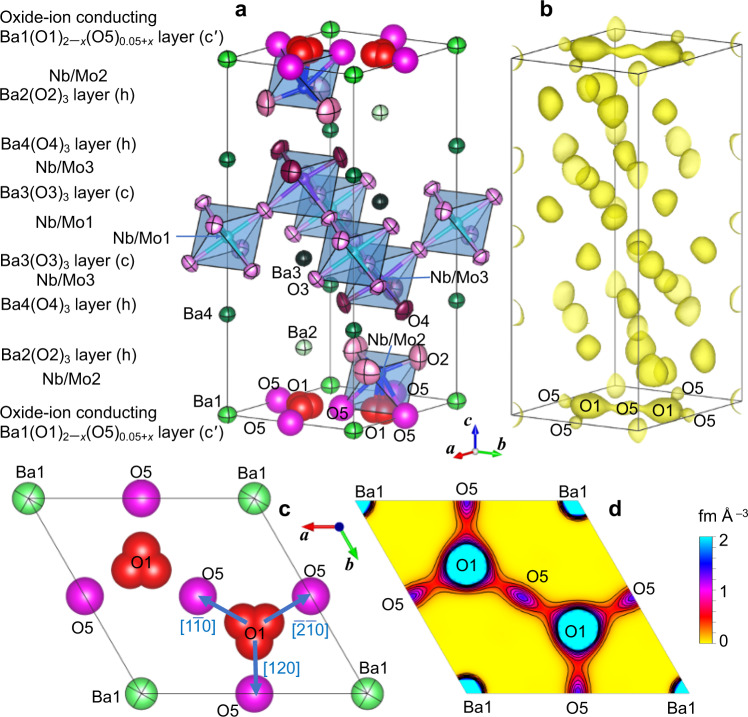
Experimental evidence of the interstitial oxygen O5 and the O1−O5 oxide-ion interstitialcy diffusion of Ba_7_Nb_3.9_Mo_1.1_O_20.05_ at a high temperature of 800 °C. **a** Refined crystal structure and **b** corresponding yellow isosurface of maximum-entropy method neutron scattering length densities (MEM NSLDs) at 0.36 fm Å^−3^ of Ba_7_Nb_3.9_Mo_1.1_O_20.05_ at 800 °C. Refined crystal structure (**c**) and corresponding MEM NSLD distribution (**d**) on the *ab* plane at *z* = 0 of Ba_7_Nb_3.9_Mo_1.1_O_20.05_ at 800 °C. In **d**, the contour lines from 0 to 2 fm Å^−3^ by the step of 0.2 fm Å^−3^. Thermal ellipsoids in panels (**a**) and (**c**) are drawn at the 90% probability level. Arrows in **c** denote the directions of oxide-ion O1-to-O5 migration.

**Fig. 3:**
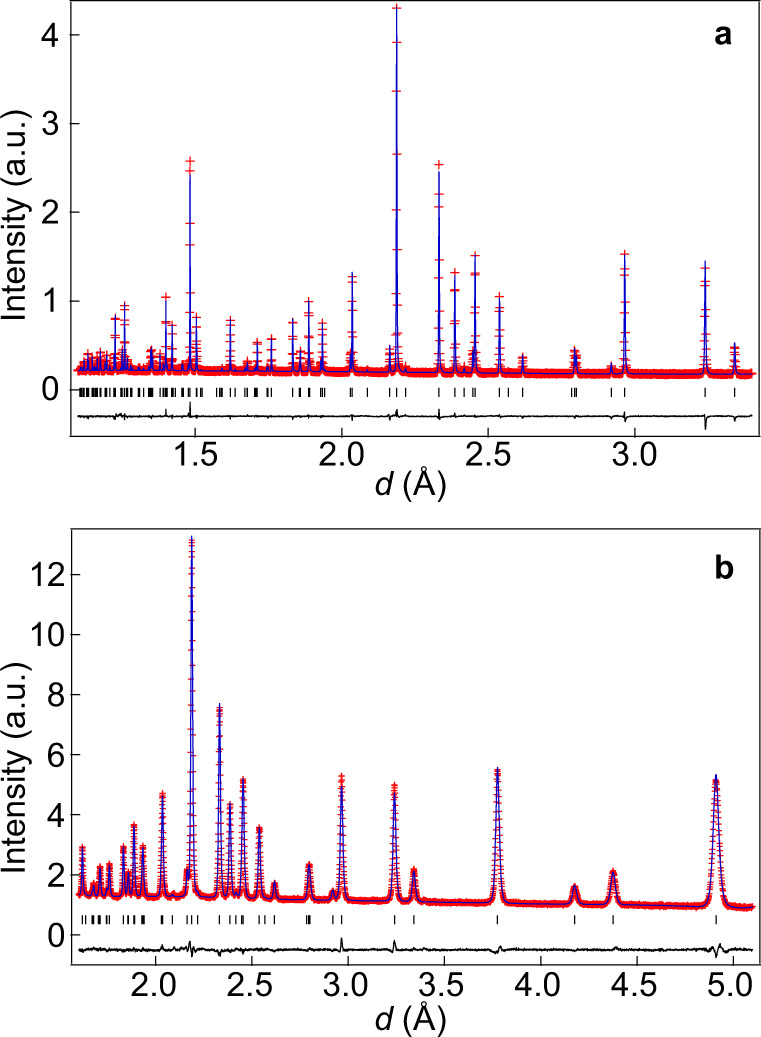
Rietveld patterns of Ba_7_Nb_3.9_Mo_1.1_O_20.05_ at 800 °C. Rietveld patterns of neutron-diffraction data taken with the (**a**) backscattering bank (*d* = 1.1−3.4 Å) and (**b**) 90^o^ bank (*d* = 1.6−5.1 Å) of the SuperHRPD diffractometer in vacuum at 800 °C. The observed and calculated intensities and difference plots are shown by red marks, blue and black solid lines, respectively. Black tick marks stand for calculated Bragg peak positions.

Next, we discuss the structural origin of the high oxide-ion conductivity of Ba_7_Nb_3.9_Mo_1.1_O_20.05_, based on its refined crystal structure and neutron scattering length density (NSLD) at 800 °C (Fig. 2). In the Rietveld refinements of the neutron-diffraction data, the Mo^6+^ and Nb^5+^ cations were assumed to be disordered, since they have quite similar neutron scattering lengths. By the trigonal $P\bar 3m1$ hexagonal perovskite polytype 7H (c′hhcchh; Fig. 2a), the crystal structure of Ba_7_Nb_3.9_Mo_1.1_O_20.05_ was successfully refined by Rietveld analyses of the neutron-diffraction data measured in situ at a temperature of 800 °C in vacuum on the super-high-resolution diffractometer, SuperHRPD^42,43^ at J-PARC, Japan (Fig. 3 and Supplementary Table 1). In order to examine the oxide-ion diffusion pathway and to validate the crystal structure of Ba_7_Nb_3.9_Mo_1.1_O_20.05_, the NSLD was analysed using the maximum-entropy method (MEM) and structure factors obtained through the Rietveld analysis. It is well known that the MEM is a powerful tool to study the structural disorder and ion-diffusion pathways in various ionic conductors^16,19,31^. Oxygen atoms were found to partially occupy the octahedral interstitial O5 site in the Ba1(O1)_2−*x*_(O5)_0.05+*x*_ layer (Fig. 2a), because (i) the Rietveld fit for the structural model with the O5 atom (weighted profile reliability factor *R*_wp_ = 2.39%) was lower than those without the O5 atom (*R*_wp_ = 2.47%) and (ii) the MEM NSLD distribution clearly shows the O5 site (Fig. 2b, d). Here the *x* in Ba1(O1)_2−*x*_(O5)_0.05+*x*_ is the vacancy content at the O1 site in the unit cell. We applied the split-atom model for the tetrahedral O1 site, because the atomic displacement parameter was quite high for the non-split-atom model and the Rietveld fit for the split-atom model (*R*_wp_ = 2.39%) was better than that for the non-split atom model (*R*_wp_ = 2.44%).

The crystal structure of Ba_7_Nb_3.9_Mo_1.1_O_20.05_ consists of an oxide-ion conducting Ba1(O1)_2−*x*_(O5)_0.05+*x*_ layer (c′), two Ba2(O2)_3_ layers (h), two Ba4(O4)_3_ layers (h), two Ba3(O3)_3_ layers (c), and Nb and Mo cations at the Nb/Mo1, Nb/Mo2 and Nb/Mo3 sites (Fig. 2a). A striking feature of the MEM NSLD distribution of Ba_7_Nb_3.9_Mo_1.1_O_20.05_ at 800 °C is the connected oxide-ion diffusional pathway between the tetrahedral O1 and interstitial octahedral O5 sites on the oxide-ion conducting Ba1(O1)_2−*x*_(O5)_0.05+*x*_ layer (c′) (Fig. 2b, d). The oxide ions two-dimensionally migrate through both lattice O1 and interstitial O5 sites, which indicates the interstitialcy mechanism of oxide-ion diffusion. The bond-valence-based energy barriers for oxide-ion migration, *E*_b_, for the refined crystal structure of Ba_7_Nb_3.9_Mo_1.1_O_20.05_ at 800 °C also supported this 2D feature, because the *E*_b_ along the *ab* plane (0.19 eV) is much lower than *E*_b_ along the *c* axis (1.54 eV). Ba_7_Nb_3.9_Mo_1.1_O_20.05_ has an excess oxygen of *x* = 0.05 (O_20+*x*_ or O_0.05_ in Ba_7_Nb_3.9_Mo_1.1_O_20.05_) compared with the mother material Ba_7_Nb_4_MoO_20_, which leads to a larger amount of interstitial oxygen and the higher oxide-ion conductivity of Ba_7_Nb_3.9_Mo_1.1_O_20.05_ (Fig. 1c).

In conclusion, we have discovered a structure family of rare-earth-free oxide-ion conductors based on the hexagonal perovskite related oxide Ba_7_Nb_4_MoO_20_. Ba_7_Nb_3.9_Mo_1.1_O_20.05_ shows a wide stability range and predominantly oxide-ion conduction in the oxygen partial pressure range from 2 × 10^–26^ to 1 atm at 600 °C. The bulk conductivity of Ba_7_Nb_3.9_Mo_1.1_O_20.05_ is as high as 5.8 × 10^−4^ S cm^−1^ at 310 °C. This high conductivity is ascribed to the interstitial O5 oxygen, 2D oxide-ion O1 − O5 diffusion through the lattice tetrahedral O1 and interstitial O5 octahedral oxygen sites on the *ab* plane at *z* = 0 and to the low activation energy for oxide-ion conductivity. The (tetrahedral O1)–(octahedral O5) diffusion pathways in Ba_7_Nb_3.9_Mo_1.1_O_20.05_ are along the [$1\bar 10$], [120] and [$\bar 2\bar 10$] directions (Arrows in Fig. 2c), which are the same as those for the (tetrahedral O3)–(octahedral O2) migration paths in the hexagonal perovskite related oxide Ba_3_MoNbO_8.5–*δ*_^31^. This strongly suggests that the (tetrahedral)–(octahedral) oxide-ion migration pathway along the [$1\bar 10$], [120] and [$\bar 2\bar 10$] directions on the oxygen deficient c′ layer is a common feature of the oxide-ion conductors with hexagonal perovskite related structures. This feature would be a guide for design of oxide-ion conductors with the hexagonal perovskite-related structures. The present finding of high oxide-ion conductivities in rare-earth-free Ba_7_Nb_3.9_Mo_1.1_O_20.05_ suggests the ability of various hexagonal perovskite related oxides as superior oxide-ion conductors.

## Methods

### Synthesis and characterization

Ba_7_Nb_3.95_Mo_1.05_O_20.025_ and Ba_7_Nb_3.9_Mo_1.1_O_20.05_ were prepared by the solid-state reactions. High-purity (> 99.9%) BaCO_3_, Nb_2_O_5_, and MoO_3_ were mixed and ground using an agate mortar and pestle as ethanol slurries and dry powders repeatedly for 0.5–2 h. The obtained mixtures were calcined at 900 °C for 10–12 h in static air. The calcined samples were crushed and ground using an agate mortar and pestle as ethanol slurries and dry powders repeatedly for 0.5–2 h. The powders thus obtained were uniaxially pressed into pellets at 62–150 MPa and subsequently sintered in static air at 1100 °C for 24 h. Parts of the sintered pellets were crushed and ground into white powders to measure X-ray powder diffraction, atomic absorption spectroscopy (AAS, Hitachi Z-2300), inductively coupled plasma optical emission spectroscopy (ICP-OES, Hitachi PS3520UVDD), and thermogravimetric (TG) data. To identify the existing phases, X-ray powder diffraction patterns of Ba_7_Nb_3.95_Mo_1.05_O_20.025_ and Ba_7_Nb_3.9_Mo_1.1_O_20.05_ were measured at RT with an X-ray powder diffractometer (BRUKER D8 Advance, Cu *K*α radiation, 2*θ* range: 5−90°). The chemical composition of Ba_7_Nb_3.9_Mo_1.1_O_20.05_ was examined by energy dispersive XRF analyses (Rigaku, NEX DE). XPS spectra of Ba_7_Nb_3.9_Mo_1.1_O_20.05_ were measured using an X-ray photoelectron spectrometer (ULVAC PHI 5000 Versa Probe III). TG analysis was carried out in dry air using a Bruker-AXS 2020SA instrument at the heating and cooling rates of 10 °C min^−1^. The heating and cooling cycle was repeated three times to negate the influence of absorbed species, such as water and to confirm the reproducibility of the measurement.

### Measurements of electrical conductivity, oxygen diffusion coefficient and transport properties

The electrical conductivities of Ba_7_Nb_3.9_Mo_1.1_O_20.05_ were measured as a function of temperature by AC impedance spectroscopy in flowing dry air, N_2_, and O_2_ gases (100 mL min^−1^) using a sintered pellet (20 mm in diameter, 2.7 mm in thickness, relative density of 100−98%) with Pt electrodes. Impedance spectra were recorded with a Solartron 1260 impedance analyser in the frequency range of 10 MHz−1 Hz at an applied alternating voltage of 100 mV. The activation energies, *E*_a_, for the conductivities were estimated using the Arrhenius equation:

1 $$\sigma = \frac{{A_0}}{T}{\mathrm{exp}}\left( { - \frac{{E_{\rm{a}}}}{{kT}}} \right)$$

where *A*_0_, *k*, and *T* are the pre-exponential factor, Boltzmann constant, and absolute temperature, respectively. Oxygen concentration cell measurements were conducted to investigate the oxygen transport number *t*_ion_ using a sintered pellet (20 mm in diameter, 4.5 mm in height, and relative density of 100−98%) attached to an alumina tube with a glass seal. One side of the pellet was exposed to flowing dry air and the other side to flowing dry O_2_ (Air/O_2_), N_2_ (Air/N_2_), or 5% H_2_ in N_2_ (Air/5% H_2_ in N_2_) gases at high temperatures. The electromotive forces of the concentration cell were recorded with a Keithley model 617 electrometer. The following Nernst equation was utilized to estimate the *t*_ion_:

2 $$E = t_{{\mathrm{ion}}}\frac{{RT}}{{4F}}{\mathrm{ln}}\left( {\frac{{p\left( {{\rm{O}}_2} \right)}}{{p^0\left( {{\rm{O}}_2} \right)}}} \right)$$

where *F* is the Faraday constant, *R* is the gas constant, *T* is the absolute temperature, *p*(O_2_) is the oxygen partial pressure of the gas of O_2_, N_2_, 5% H_2_ in N_2_, and *p*^0^(O_2_) (= 0.21 atm) is the oxygen partial pressure of dry air. After the oxygen concentration cell measurements, the surface of the pellet was ground with sandpaper carefully to remove the Pt paste and then crushed and ground into powder. X-ray diffraction patterns of the resulting powders were measured to investigate the phase stability at high temperatures and different atmospheres.

The total electrical conductivity *σ*_tot_ of the Ba_7_Nb_3.9_Mo_1.1_O_20.05_ pellet (relative density: 95%) was measured by a DC-4-probe method with Pt electrodes at various oxygen partial pressure *p*(O_2_). The *p*(O_2_) was controlled using a mixture of O_2_, N_2_, and 5% H_2_ in N_2_ and *p*(O_2_) was monitored by an oxygen sensor.

^18^O tracer diffusion measurements of dense Ba_7_Nb_3.9_Mo_1.1_O_20.05_ pellets (relative density of 98-100%) were carried out using the line scan method by secondary ion mass spectrometry (SIMS)^41^. Each sample prepared was polished with diamond spray media down to a finish of 0.25 µm. Samples were pre-annealed in dry research grade oxygen (BOC 99.996%) of natural isotopic abundance for a duration of 10 times that of the isotopic exchange. The samples were subsequently annealed for 2 h in ^18^O-enriched gas at a pressure of ≃200 mbar. After the exchange anneal, the samples were cut perpendicular to the original surface and the exposed cross-sections polished to 0.25 μm finish, as above. The oxygen diffusion profiles were measured by Time-of-Flight Secondary Ion Mass Spectrometry (ToF-SIMS) using a ToF-SIMS.5 instrument (IONTOF GmbH) using Bi^+^ ions at 25 keV energy. Values of oxygen self-diffusion, D*, and surface exchange, *k*, coefficients were obtained by fitting the experimental data to Crank’s solution of Fick’s 2^nd^ law of diffusion^41,44^ using the TraceX software^45^. The microstructure of the Ba_7_Nb_3.9_Mo_1.1_O_20.05_ pellet used for the ^18^O tracer diffusion measurements was observed by a scanning electron microscope (KEYENCE VE-8800).

### Neutron-diffraction measurements of Ba_7_Nb_3.9_Mo_1.1_O_20.05_ at 800 °C, Rietveld and MEM analyses

High-temperature neutron-diffraction measurements of Ba_7_Nb_3.9_Mo_1.1_O_20.05_ pellets (8.7 mm in diameter, 43 mm in height) in a Ti-Zr alloy holder were carried out in vacuum using a super-high-resolution time-of-flight (TOF) neutron diffractometer (SuperHRPD) installed at the Materials and Life Science Experimental Facility of J-PARC, Japan^42,43^. The absorption correction was performed using the method given by Rouse and Cooper^46^. The diffraction data were analysed by the Rietveld method using the Z-Rietveld program^47^. The neutron scattering length density distribution was investigated using the MEM. The MEM analysis was carried out with computer program, Dysnomia^48^, using the structure factors obtained in the Rietveld refinement of the neutron diffraction data at 800 °C. The MEM calculations were performed with the unit cell divided into 60 × 60 × 168 pixels.

### Computation of the bond-valence-based energy barrier for oxide-ion migration

The bond-valence-based energy landscapes for a test oxide ion were calculated using the SoftBV^49^ software with a spatial resolution of 0.1 Å.

The refined crystal structure, MEM neutron scattering length density distributions, and bond-valence-based energy landscape were depicted using VESTA^50^.

## Supplementary information

Supplementary Information

## Data Availability

The data that support the findings of this study are available from the corresponding author upon reasonable request.
