# Supplementary material for: High oxide-ion conductivity through the interstitial oxygen site in Ba7Nb4MoO20-based hexagonal perovskite related oxides
Source: Nat Commun. 2021 Jan 25;12:556. doi: 10.1038/s41467-020-20859-w (PMC7835212; doi:10.1038/s41467-020-20859-w)
Supplement: Supplementary file 1 — Supplementary Information [file 41467_2020_20859_MOESM1_ESM.docx]

**Supplementary information**

**High Oxide-Ion Conductivity through the Interstitial Oxygen Site**

**in Ba_7_Nb_4_MoO_20_-Based Hexagonal Perovskite Related Oxides**

Yashima M. et al.

**Supplementary Figures**


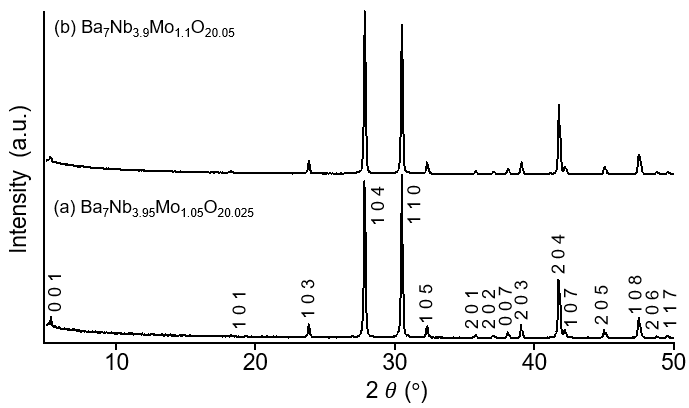


**Supplementary Figure 1. X-ray powder diffraction patterns of (a) Ba_7_Nb_3.95_Mo_1.05_O_20.025_ and (b) Ba_7_Nb_3.9_Mo_1.1_O_20.05_.** *hkl* denotes the reflection index of the trigonal phase with the $P\bar{3}m1$ hexagonal perovskite related structure.^1^ For both compositions, the 001 reflection is observed, which is a signature of 7H hexagonal perovskite.


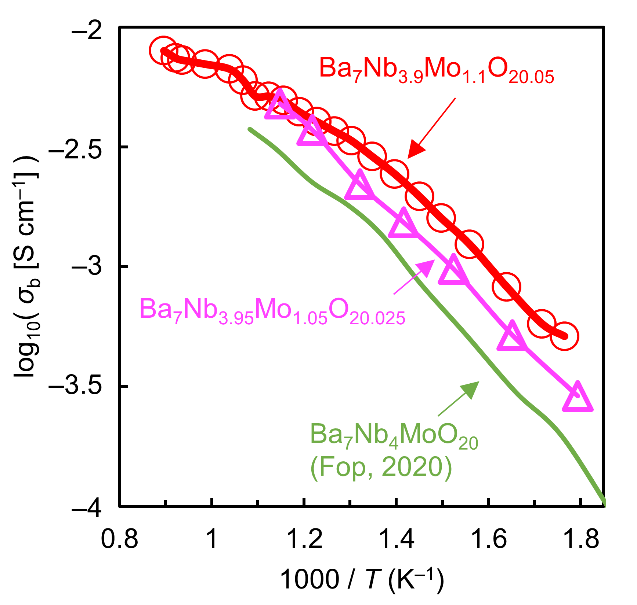


**Supplementary Figure 2. Arrhenius plots of bulk conductivity *σ*_b_ of Ba_7_Nb_4_MoO_20_ (reported by Fop et al. in 2020),**^2^ **Ba_7_Nb_3.95_Mo_1.05_O_20.025_, and Ba_7_Nb_3.9_Mo_1.1_O_20.05_.** Impedance analysis of Ba_7_Nb_3.9_Mo_1.1_O_20.05_ is shown in Figure 1a,b. Impedance analysis of Ba_7_Nb_3.95_Mo_1.05_O_20.025_ is shown in Supplementary Figure 6.

**
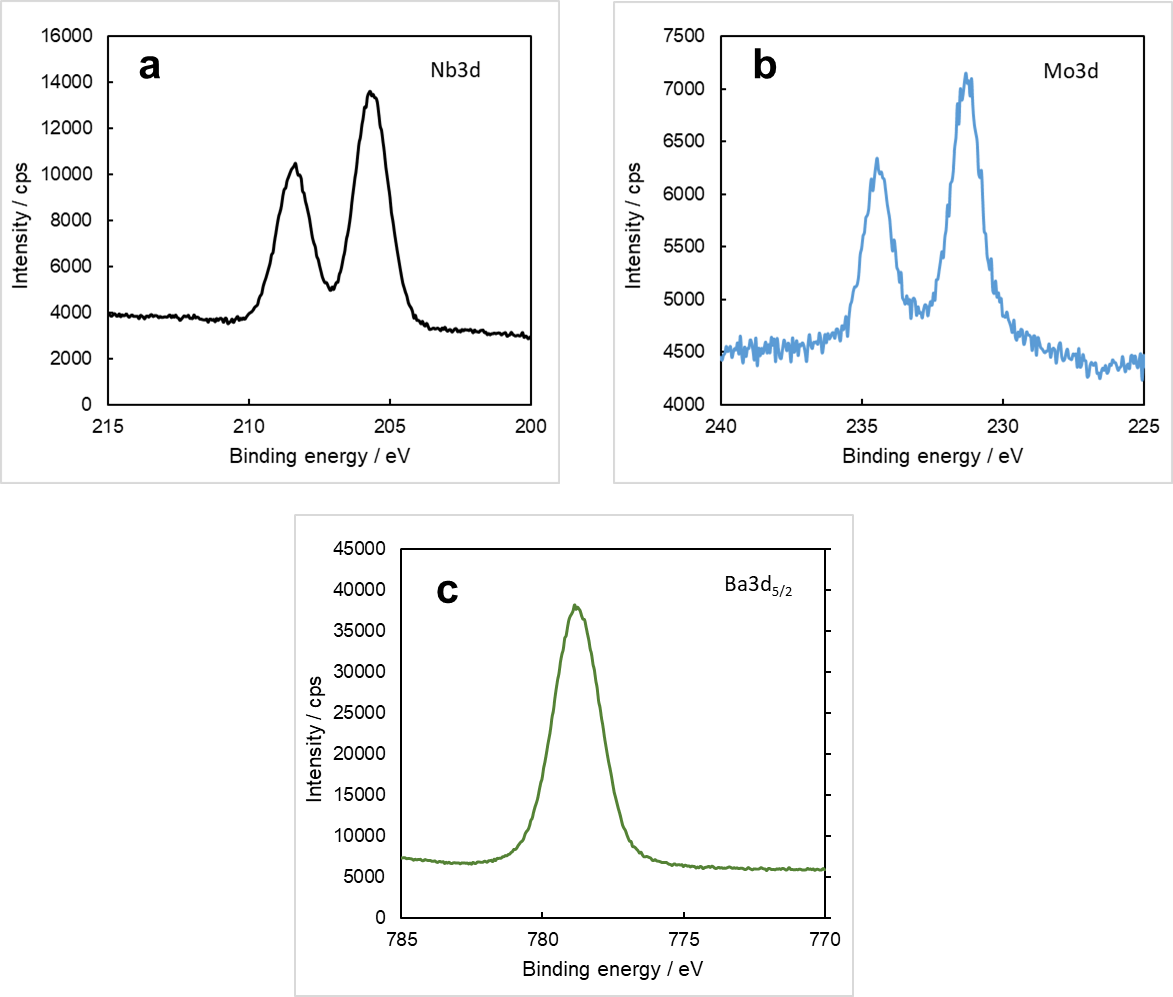
**

**Supplementary Figure 3. XPS spectra of (a) Nb 3d, (b) Mo 3d and (c) Ba 3d in Ba_7_Nb_3.9_Mo_1.1_O_20.05_.** Two peaks at 205.7 and 208.4 eV in (a) represent the Nb^5+^ 3d_5/2_ and Nb^5+^ 3d_3/2_ components, respectively. Two peaks at 231.3 and 234.5 eV in (b) indicate the Mo^6+^ 3d_5/2_ and Mo^6+^ 3d_3/2_ components, respectively. The peak at 778.8 eV in (c) represents the Ba^2+^ 3d_5/2_ component. Literature^3-10^ was referred to for the peak identification.

**Supplementary Figure 4. Thermogravimetric data of a Ba_7_Nb_3.9_Mo_1.1_O_20.05_ sample performed in dry air on (a) the 1st cycle and (b) the 2nd and 3rd cycles.** Heating and cooling rates were 5 ^o^C min^−1^. The small weight loss suggests small amounts of oxygen deficiency and/or water desorption. In this paper, we describe the composition as Ba_7_Nb_3.9_Mo_1.1_O_20.05_ for simplicity. Smaller weight loss of Ba_7_Nb_3.9_Mo_1.1_O_20.05_ than that of Ba_3_MoNbO_8.5–_*_δ_*^11^ suggests the higher stability of Ba_7_Nb_3.9_Mo_1.1_O_20.05_ at high temperatures.

**
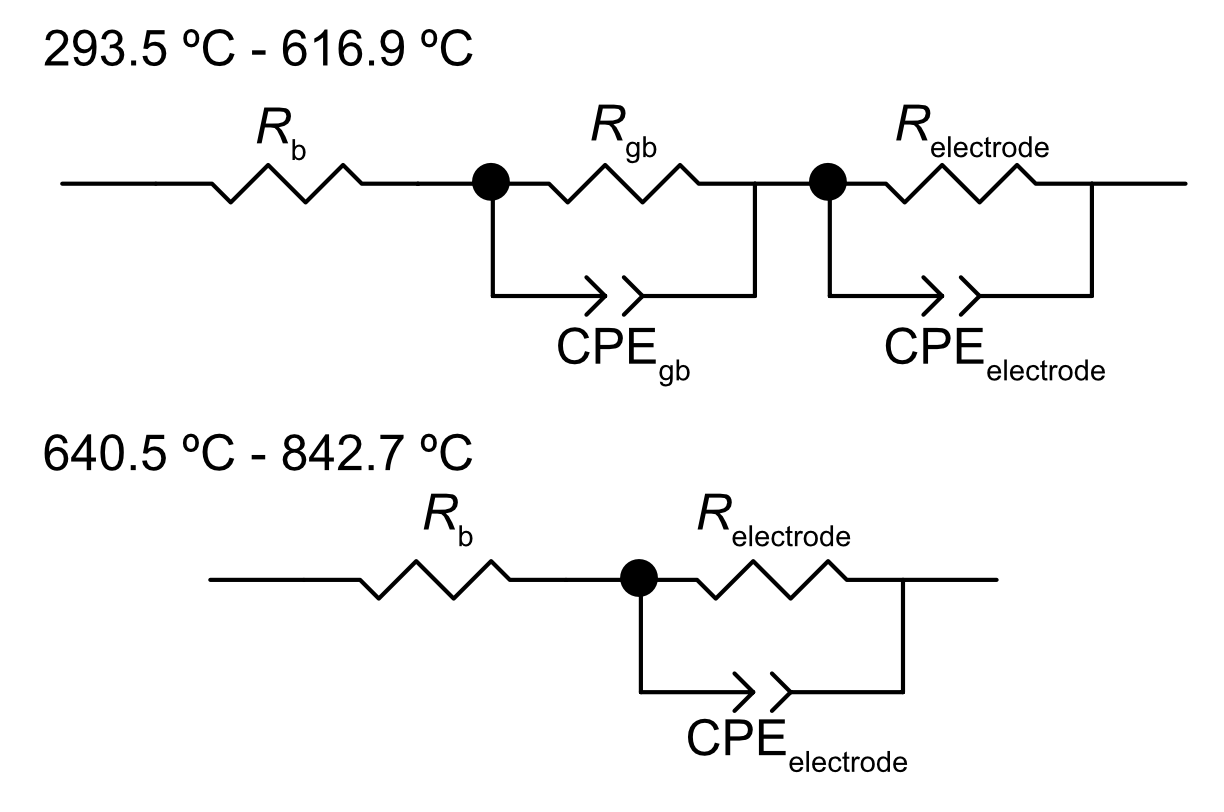
**

**Supplementary Figure 5.** **Equivalent circuits used to model the impedance data of Ba_7_Nb_3.9_Mo_1.1_O_20.05_.** *R* and CPE denote a resistor and a constant phase element, respectively, where the subscripts b and gb stand for the bulk and grain boundary, respectively. The fitting for the data of Ba_7_Nb_3.9_Mo_1.1_O_20.05_ at 616.9 ºC gave the values of *σ*_b_ = 5.1×10^–3^ S cm^–1^, *σ*_gb_ = 1.5×10^–2^ S cm^–1^，and *C*_gb_ ≃ 1.1 nF cm^–1^. ﻿With increasing temperature, the magnitudes of the grain boundary conductivity increase more rapidly than those of bulk conductivity. These results suggest that the grain boundary is more conductive than the grains above 630 ºC. ﻿Therefore, the intercept on the *Z*′ axis can be treated as the bulk resistivity, because *R*_gb_ is much less than *R*_b_ and *R*_gb_ + *R*_b_ ≈ *R*_b_.^12^

^
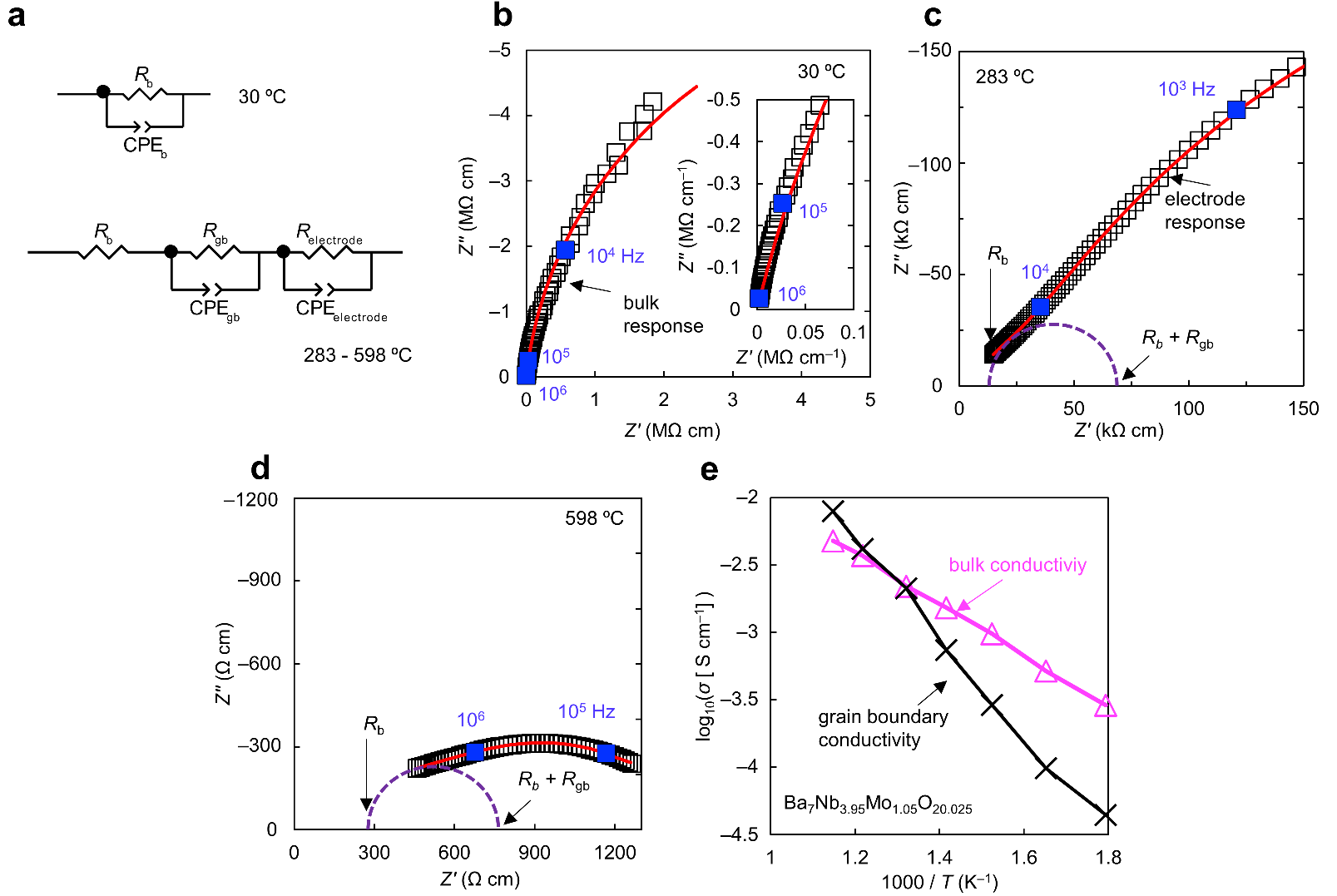
^

**Supplementary Figure 6.** **Impedance analysis of Ba_7_Nb_3.95_Mo_1.05_O_20.025_.** (a) Equivalent circuits for data analysis. (b-d) Complex impedance plots of Ba_7_Nb_3.95_Mo_1.05_O_20.025_ measured at (b) 30, (c) 283, and (d) 598 ºC. (e) Arrhenius plots of bulk (pink) and grain-boundary (black) conductivities of Ba_7_Nb_3.95_Mo_1.05_O_20.025_.


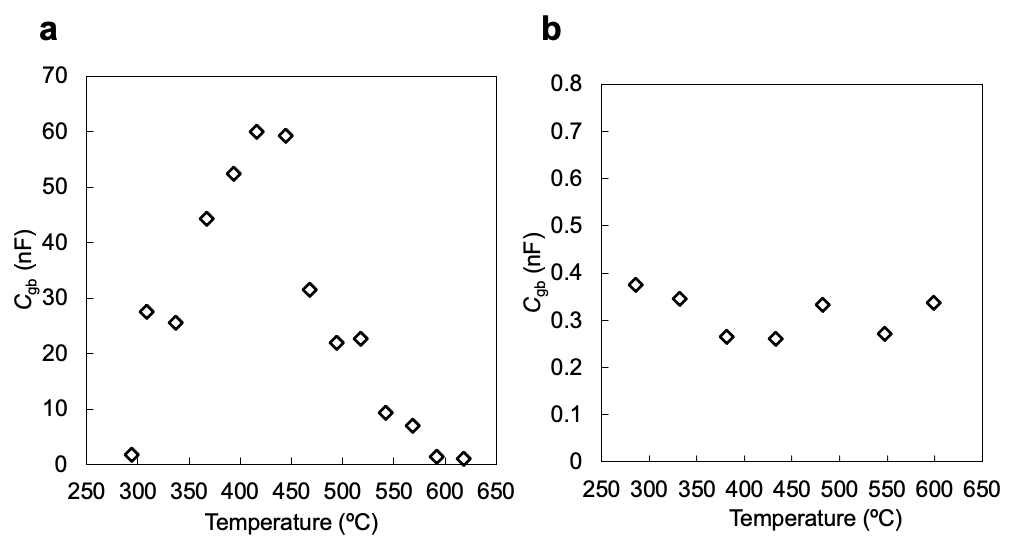


**Supplementary Figure 7.** **Temperature dependencies of the grain-boundary capacitance *C*_gb_ of (a) Ba_7_Nb_3.9_Mo_1.1_O_20.05_ and (b) Ba_7_Nb_3.95_Mo_1.05_O_20.025_.**


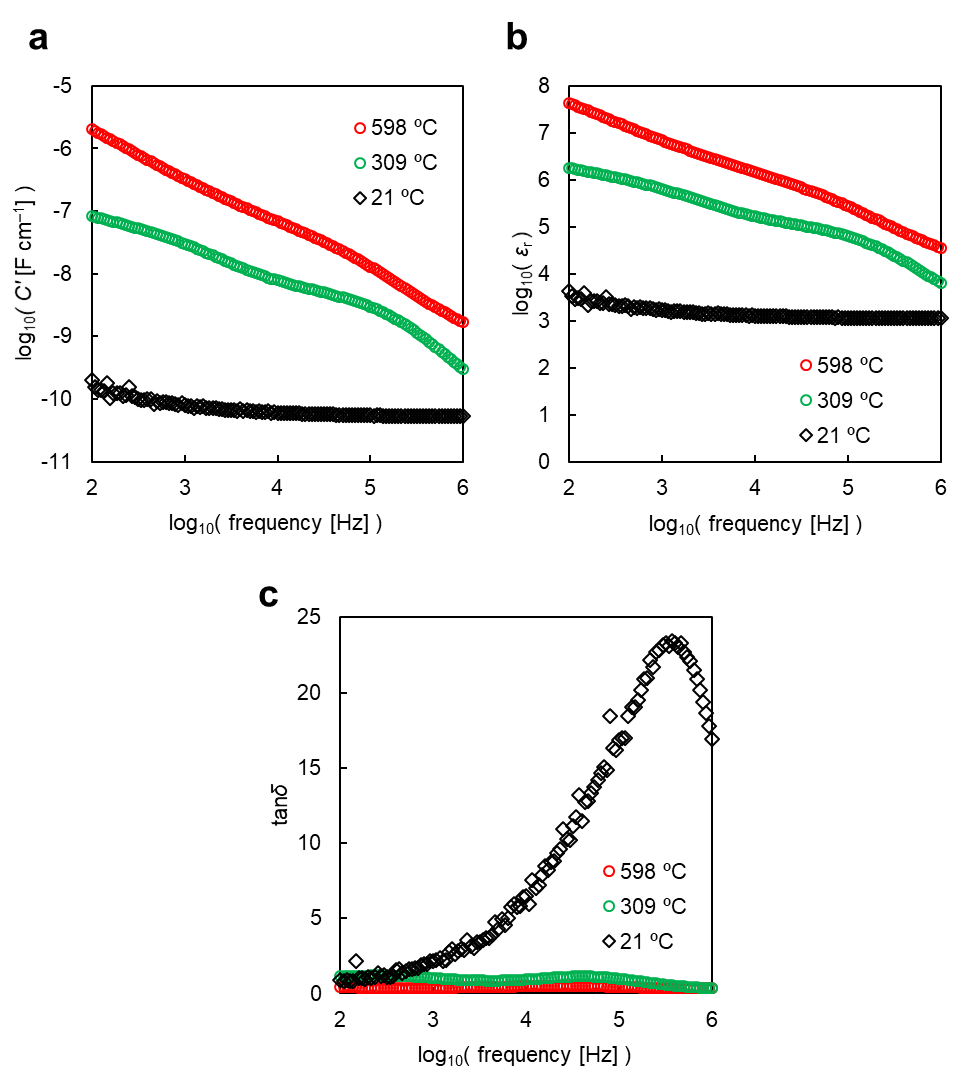


**Supplementary Figure 8.** **Frequency dependencies of (a) capacitance *C'*, (b) dielectric constant (relative permittivity) *ε*_r_, and loss tangent tan*δ* of Ba_7_Nb_3.9_Mo_1.1_O_20.05_.**


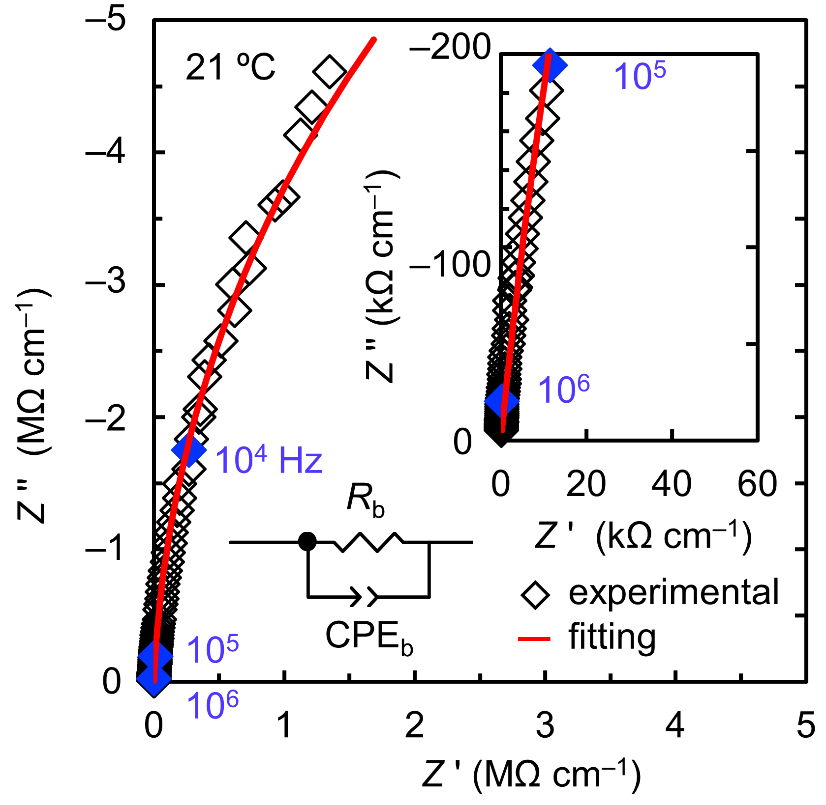


**Supplementary Figure 9. Bulk conductivity of Ba_7_Nb_3.9_Mo_1.1_O_20.05_ at 21 ºC.** Complex impedance plot of Ba_7_Nb_3.9_Mo_1.1_O_20.05_ recorded in dry air at 21 ºC. The fitting for the data of Ba_7_Nb_3.9_Mo_1.1_O_20.05_ gave *σ*_b_ = 6.32(19)×10^–8^ S cm^–1^ and *C*_b_ ≃ 10.2(3) pF cm^–1^.


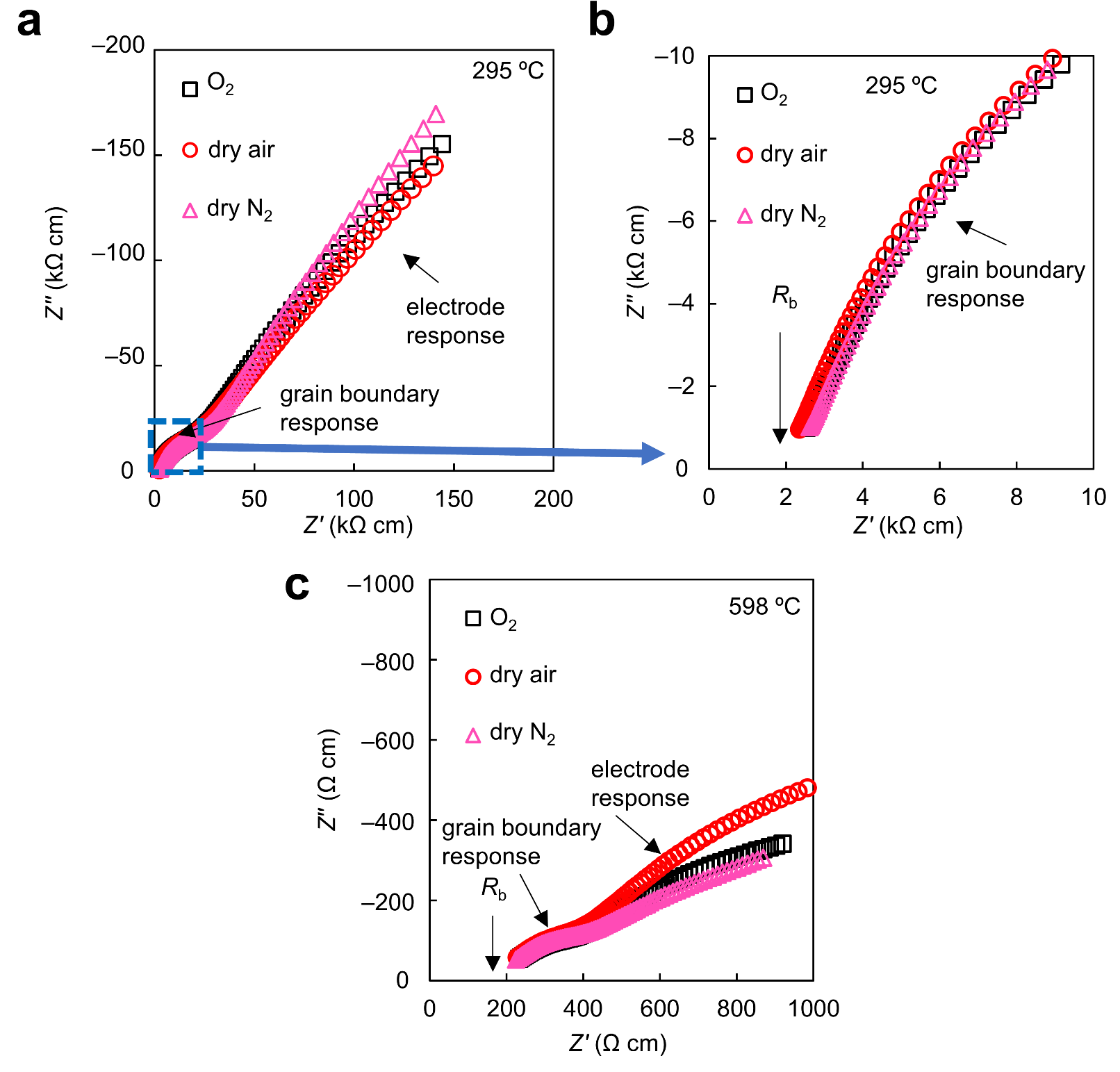


**Supplementary Figure. 10 ﻿Complex impedance plane plots of Ba_7_Nb_3.9_Mo_1.1_O_20.05_ recorded under different atmospheres.** (a) Complex impedance plots at 295 ºC, (b) expanded scale for the high frequency data, and (c) Complex impedance plots at 598 ºC under O_2_, dry air, and N_2_ flowing.


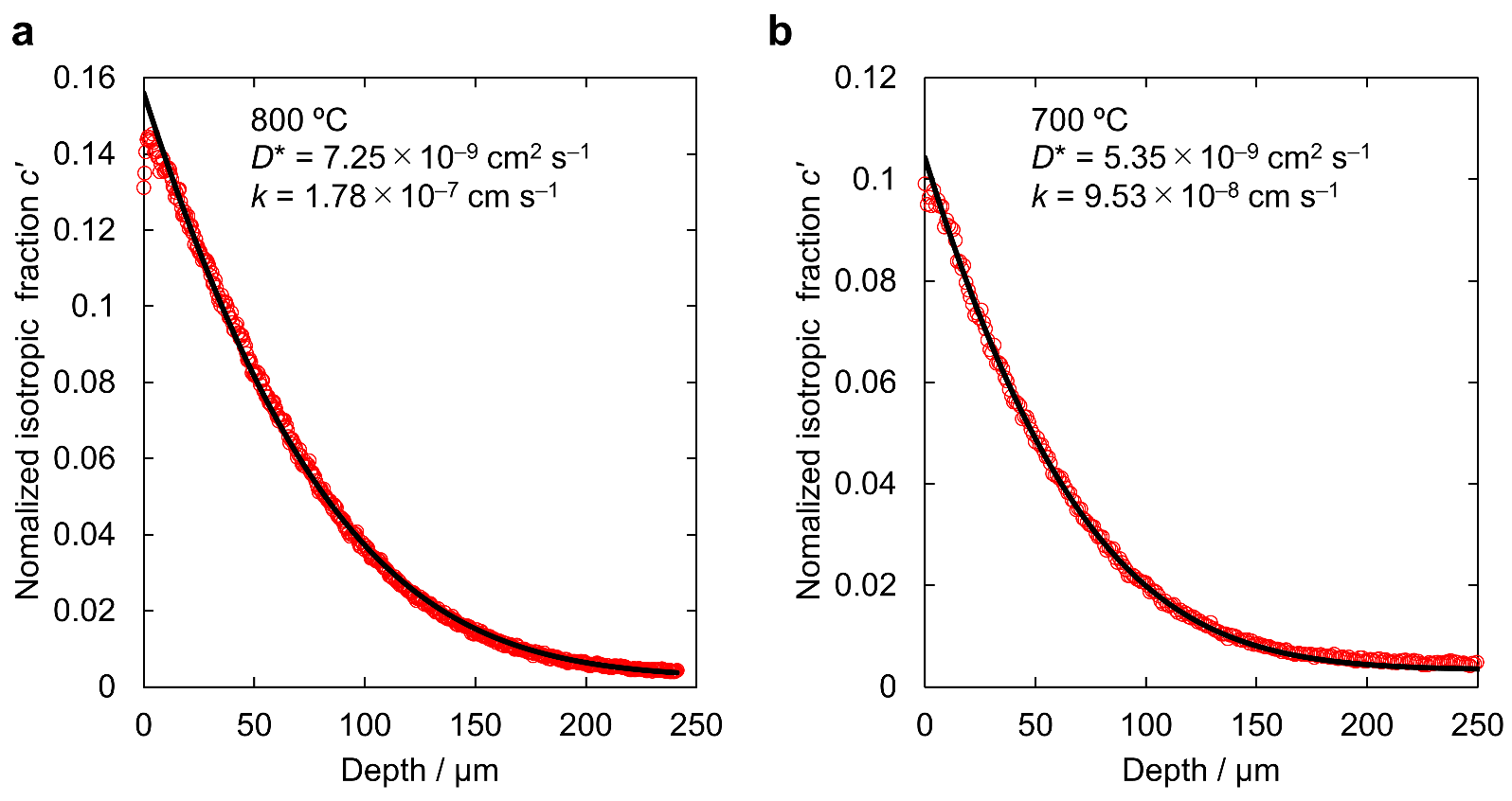


**Supplementary Figure 11.** **﻿^18^O tracer diffusion profiles for Ba_7_Nb_3.9_Mo_1.1_O_20.05_.** Profiles after the exchanges at (a) 800ºC and (b) 700 ºC for ≃ 7200 s with *p*(^18^O_2_) ≃ 179 mbar. Red circles denote experimental data. Black lines denote the lines of best fit.


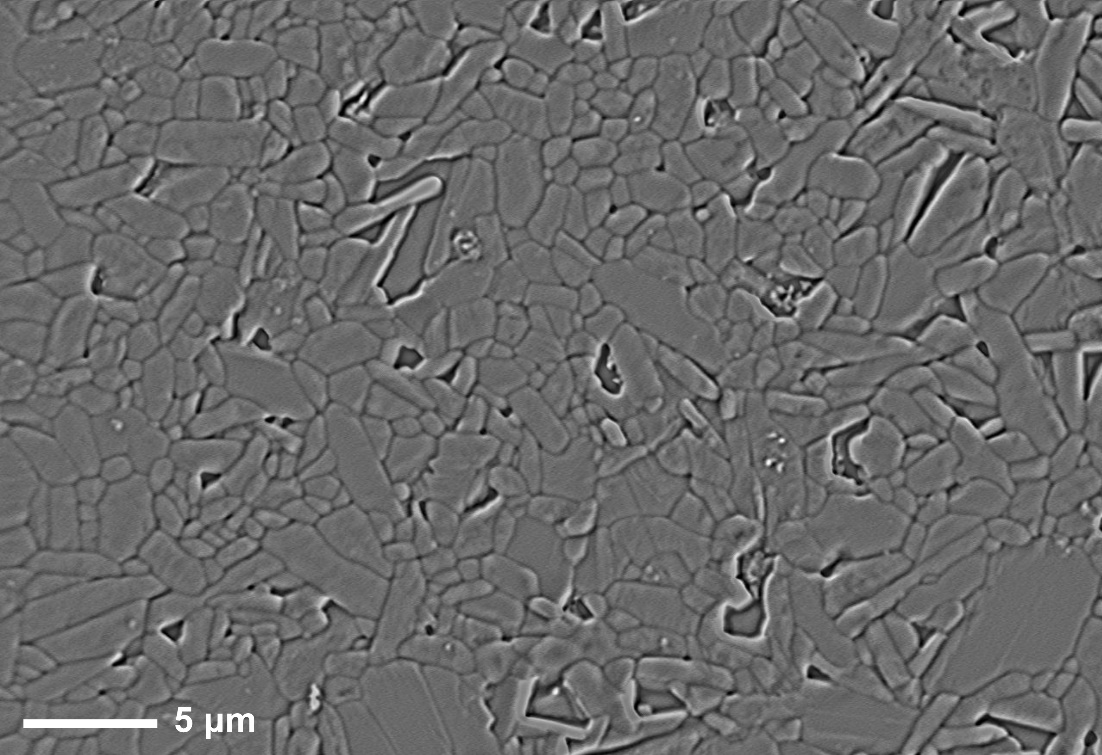


**Supplementary Figure 12.** **﻿** **SEM micrograph of Ba_7_Nb_3.9_Mo_1.1_O_20.05_ used in the ^18^O tracer diffusion experiment.** The sample was polished and thermally etched at 1150 ºC for 2 h prior to the SEM observation.

**
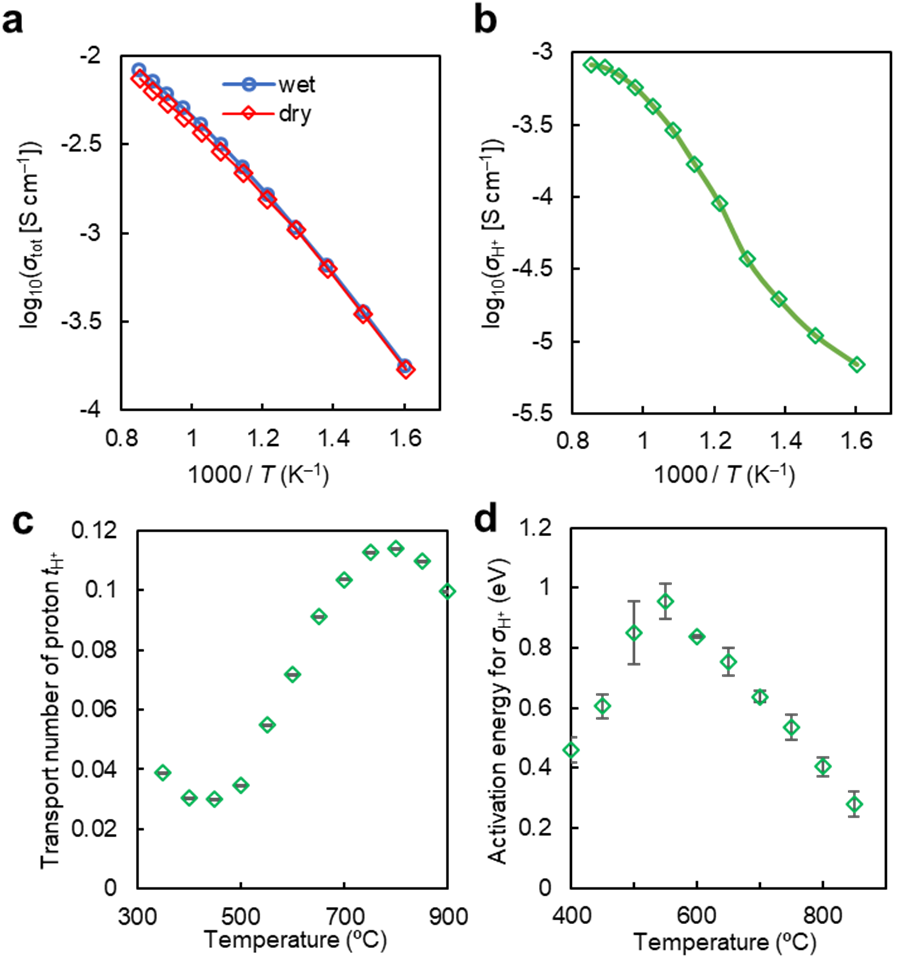
**

**Supplementary Figure 13. Proton conduction of Ba_7_Nb_3.9_Mo_1.1_O_20.05_ in wet air.** (a) Arrhenius plots of total DC electrical conductivities of Ba_7_Nb_3.9_Mo_1.1_O_20.05_ in dry air *σ*_dry_ (*p*(H_2_O) < 2 × 10^–4^ atm; red line and diamonds) and in wet air *σ*_wet_ (*p*(H_2_O) = 2.3 × 10^–2^ atm; blue line and circles). (b) Arrhenius plot of proton conductivity *σ*_H⁺_ of Ba_7_Nb_3.9_Mo_1.1_O_20.05_ in wet air. Here the *σ*_H⁺_ was estimated using the equation *σ*_H⁺_ = *σ*_wet_ – *σ*_dry_. (c) Transport number of proton *t*_H⁺_ for Ba_7_Nb_3.9_Mo_1.1_O_20.05_, which was estimated using the equation *t*_H⁺_ = *σ*_H⁺_ / *σ*_wet_. The *t*_H⁺_ increases with temperature from 450 to 800 ^o^C. (d) Activation energy for *σ*_H⁺_ of Ba_7_Nb_3.9_Mo_1.1_O_20.05_, which increases between 400 and 550 ^o^C, while decreases with an increase of temperature between 550 and 850 ^o^C. Strictly speaking, Ba_7_Nb_3.9_Mo_1.1_O_20.05_ is a mixed oxide-ion and proton conductor. Since the proton transport number is small (less than 12%; Supplementary Fig. 7c), the oxide ion is the dominant carrier and Ba_7_Nb_3.9_Mo_1.1_O_20.05_ is an oxide-ion conductor. The proton conduction was confirmed also in the bulk and grain boundary conductivities. Error bars in panels (c) and (d) are the estimated standard deviations.


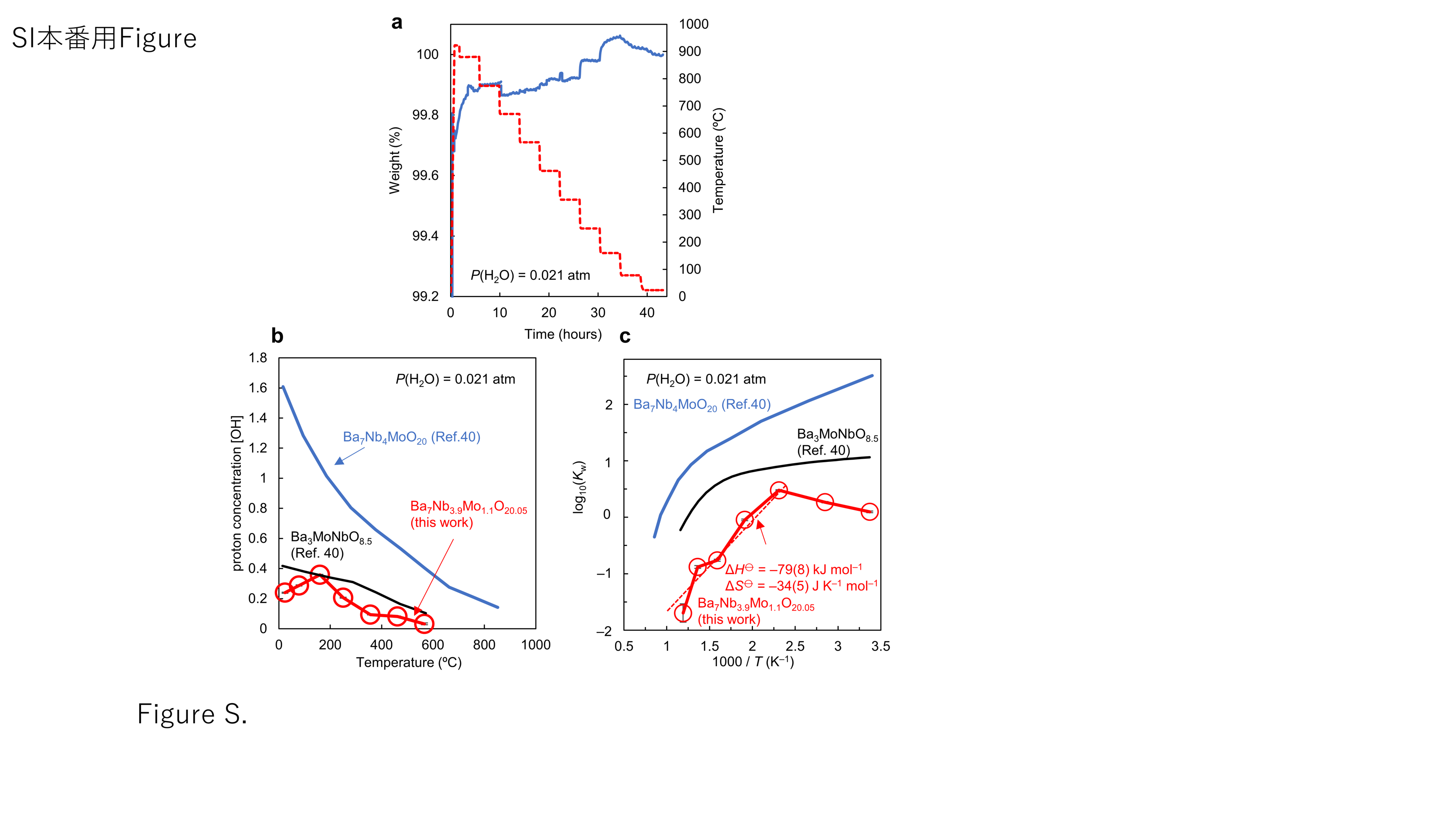


**Supplementary Figure 14.** Water incorporation of Ba_7_Nb_3.9_Mo_1.1_O_20.05_. (a) Weight change of Ba_7_Nb_3.9_Mo_1.1_O_20.05_・*y* H_2_O and temperature as a function of time in wet air (*P*(H_2_O) = 0.021 atm). (b) The proton concentration [OH] in Ba_7_Nb_3.9_Mo_1.1_O_20.05_・*y* H_2_O was calculated from thermogravimetric analysis assuming that the weight gain was ascribed to the incorporation of water. The incorporated water amount, *y,* was ≃ 0.12 at room temperature. The data for Ba_7_Nb_4_MoO_20_ and Ba_3_MoNbO_8.5_ were obtained from Fop et al.^2^ (c) The calculated equilibrium constant *K*_w_ for the water incorporation reaction. Derivation and definition of *K*_w_ are discussed in Supplementary Note 2.


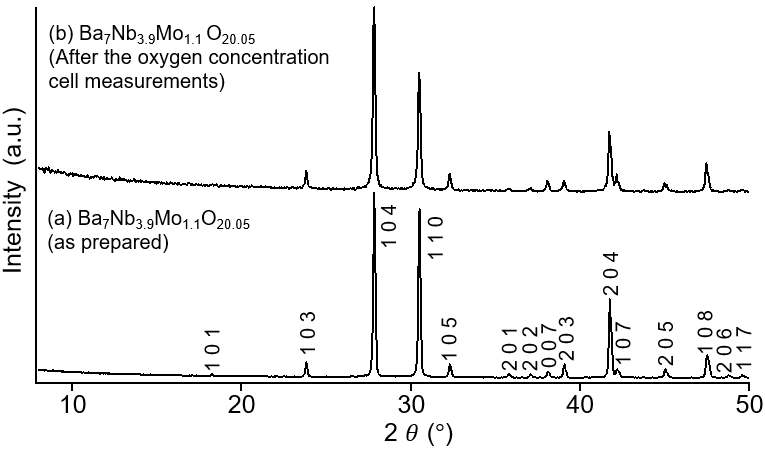


**Supplementary Figure 15.** **X-ray powder diffraction (XRD) patterns of (a) as prepared sample of Ba_7_Nb_3.9_Mo_1.1_O_20.05_, and (b) Ba_7_Nb_3.9_Mo_1.1_O_20.05_ sample after the oxygen concentration cell measurements (5% H_2_ in N_2_/air and O_2_/air).** Both XRD patterns are similar, which indicates the high phase stability of Ba_7_Nb_3.9_Mo_1.1_O_20.05_. The measured *P*(O_2_) of 5% H_2_ in N_2_ was 1.2 × 10^−27^ atm, thus, Ba_7_Nb_3.9_Mo_1.1_O_20.05_ exhibits high phase stability in the wide *P*(O_2_) region between *P*(O_2_) = 1.2 × 10^−27^ and 1 atm.

**Supplementary Table 1.** Refined Crystallographic Parameters and Reliability Factors in the Rietveld Analysis of the Neutron Diffraction Data of Ba_7_Nb_3.9_Mo_1.1_O_20.05_ Measured at 800 ºC.

| site *s*  atom label | atom *X* | site^¶^ | *x* | *y* | *z* | *g*(*X*; *s*) | *U*_eq_ or *U*_iso_  / Å^2^ | *U*_11_ / Å^2^ | | *U*_22_ / Å^2^ | *U*_33_ / Å^2^ | *U*_12_ / Å^2^ | *U*_13_ / Å^2^ | *U*_23_ / Å^2^ | BVS |
| --- | --- | --- | --- | --- | --- | --- | --- | --- | --- | --- | --- | --- | --- | --- | --- |
| Ba1 | Ba | 1*a* | 0 | 0 | 0 | 1 | 0.0254(7) | 0.0267(9) | | 0.0267(9) | 0.0228(17) | 0.0133(5) | 0 | 0 | 1.93 |
| Ba2 | Ba | 2*d* | 1/3 | 2/3 | 0.82350(6) | 1 | 0.0197(2) |  | |  |  |  |  |  | 2.04 |
| Ba3 | Ba | 2*d* | 1/3 | 2/3 | 0.57329(8) | 1 | 0.0197(2) |  | |  |  |  |  |  | 2.17 |
| Ba4 | Ba | 2*c* | 0 | 0 | 0.28211(8) | 1 | 0.0197(2) |  | |  |  |  |  |  | 1.76 |
| Mo/Nb1 | Nb_0.78_Mo_0.22_ | 1*b* | 0 | 0 | 1/2 | 1 | 0.01310(13) | |  |  |  |  |  |  | 4.33 |
| Mo/Nb2 | Nb_0.78_Mo_0.22_ | 2*d* | 1/3 | 2/3 | 0.09369(5) | 1 | 0.01310(13) | |  |  |  |  |  |  | 5.51 |
| Mo/Nb3 | Nb_0.78_Mo_0.22_ | 2*d* | 1/3 | 2/3 | 0.34940(5) | 1 | 0.01310(13) | |  |  |  |  |  |  | 4.57 |
| O1 | O | 6*i* | 0.3616 (5) | 0.7232(11) | –0.01132(7) | 0.3152(7) | 0.0548(13) |  | |  |  |  |  |  | 1.89 |
| O2 | O | 6*i* | 0.16848(16) | –0.16848(16) | 0.12997(5) | 1 | 0.0541(3) | 0.0534(5) | | 0.0534(5) | 0.0554(6) | 0.0407(5) | –0.0003(3) | 0.0003(3) | 1.99 |
| O3 | O | 6*i* | 0.16328(12) | –0.16358(17) | 0.43177(4) | 1 | 0.0234(3) | 0.0230(4) | | 0.0230(4) | 0.0243(5) | 0.0127(4) | 0.0038(3) | –0.0038(3) | 1.78 |
| O4 | O | 6*i* | 0.49295(10) | –0.49295(10) | 0.29512(4) | 1 | 0.0326(3) | 0.0300(4) | | 0.0300(4) | 0.0376(5) | 0.0225(5) | 0.00445(19) | –0.00445(19) | 1.99 |
| O5 | O | 3*e* | 1/2 | 0 | 0 | 0.0530(14) | 0.0548(13) |  | |  |  |  |  |  | 1.08 |

*Crystal system: trigonal. Space group: *P*$\bar{3}$*m*1 (No.164, setting 1). Lattice parameters: *a* = 5.930754(2) Å, *c* = 16.70229(16) Å. Number of formula per unit cell: *Z* = 1. *g*(*X*; *s*): Occupancy factor of *X* atom at the *s* site. *g*(Ba; Ba1) = *g*(Ba; Ba2) = *g*(Ba; Ba3) = *g*(Ba; Ba4) = *g*(Nb_0.78_Mo_0.22_; Mo/Nb1) = *g*(Nb_0.78_Mo_0.22_; Mo/Nb2) = *g*(Nb_0.78_Mo_0.22_; Mo/Nb3) = *g*(O; O2) = *g*(O; O3) = *g*(O; O4) = 1; 2*g*(O1) + 3*g*(O5) = 2.05. ^¶^Wyckoff Position.
*U*_iso_(*Xn*) Isotropic atomic displacement parameter of *X* atom at the *Xn* site. Linear constraints in the Rietveld analysis: *U*_iso_(Ba2) = *U*_iso_(Ba3) = *U*_iso_(Ba4), *U*_iso_(Mo/Nb1) = *U*_iso_(Mo/Nb2) = *U*_iso_(Mo/Nb3), *U*_iso_(O1) = *U*_iso_(O5).

Reliability (*R*) factors for the neutron diffraction data taken with both backscattering and 90^o^ banks: weighted profile *R* factor *R*_wp_ = 2.26%. *R* factors for the neutron diffraction data taken with backscatterng bank: *R*_wp_ = 2.39%, profile *R* factor *R*_p_ = 1.70%, *R* factor based on the Bragg intensities *R*_B_ = 4.44%, *R* factor based on the structure factors *R_F_* = 5.14%. Reliability factors for the neutron diffraction data taken with 90^o^ bank: *R*_wp_ = 2.11%, *R*_p_ = 1.64%, *R*_B_ = 2.12%, *R_F_* = 2.04%.

** BVS: Bond valence sums. Here the bond valence parameters after Gagné and Hawthorne^13^ were used for the calculations of BVSs.

**Supplementary Note 1. Structure Similarity and Bond-Valence-Based Energy Barrier for Oxide-Ion Migration of Ba_7_Nb_4_MoO_20_ and Ba_3_MoNbO_8.5–_*_δ_***

Energy barrier for oxide-ion migration of Ba_7_Nb_4_MoO_20_ and Ba_3_MoNbO_8.5–_*_δ_* were estimated using the bond-valence-based energy landscapes (BVELs) calculated for the crystallographic data in the literature. The BVELs were calculated with SoftBV program^14^ and then the energy barrier for oxide-ion migration *E*_b_ was estimated from each BVEL. The *E*_b_ value of Ba_7_Nb_4_MoO_20_, 0.21 eV, was lower than those of Ba_3_MoNbO_8.5–_*_δ_* (0.40, 0.35 and 0.33 eV using the crystallographic data reported by Yashima *et al.*,^11^ Fop *et al.*^15^ and Auckett *et al.*,^16^ respectively).

**Supplementary Note 2. The equilibrium constant for water incorporation reaction, *K*_w_.**

Similar to Fop’s report,^2^ the equilibrium constant for the water incorporation reaction *K*_w_ was calculated using extended Kröger-Vink notation.^17^ It is assumed that hydration of Ba_7_Nb_3.9_Mo_1.1_O_20.05_ occurs on two Ba2(O2)_3_ layers and an oxide-ion conducting

Ba1(O1)_2–_*_x_*O5_0.05+_*_x_* layer, called the palmierite-like layer in Fop’s report.^2^ Chemical composition of palmierite-like layers can be written Ba_3_O_8.05_□_0.95_ in Ba_7_Nb_3.9_Mo_1.1_O_20.05_, where □ is an inherent oxygen vacancy. By extended Kröger-Vink notation, the oxide ion and its effective charge can be written as $O_{O_{\frac{8.05}{9}}}^{\frac{1.95}{9} '}$. Therefore, the reaction of water incorporation is written as:

$H_{2}O(g)+O_{O_{\frac{8.05}{9}}}^{\frac{1.95}{9} '}+V_{O_{\frac{8.05}{9}}}^{\frac{16.1}{9}●}\rightleftharpoons2\mathrm{OH}_{O_{\frac{8.05}{9}}}^{\frac{7.1}{9}●}$.

With electroneutrality and site occupancy conditions,^2^ finally, equilibrium constant in terms of proton concentration [OH] is:

$K_{w}=\frac{4\left[ \mathrm{OH}_{O_{\frac{8.05}{9}}}^{\frac{7.1}{9}●} \right]^{2}}{p\left( H_{2}O \right)\left( \left[ \mathrm{OH}_{O_{\frac{8.05}{9}}}^{\frac{7.1}{9}●} \right]^{2}-18\left[ \mathrm{OH}_{O_{\frac{8.05}{9}}}^{\frac{7.1}{9}●} \right]+31.9525 \right)}$.

The regression line applied to the water incorporation data was used to estimate the standard hydration enthalpy (Δ*H*^⦵^) and entropy (Δ*S*^⦵^)^18^ according to the Van’t Hoff relation

$$RT\ln K_{w}=T\Delta S^{⦵} - \Delta H^{⦵}$$

where *R* and *T* are the gas constant and absolute temperature, respectively.

**Supplementary References**

1. García-González, E., Parras, M., & González-Calbet, J. M. Crystal structure of an unusual polytype: 7H-Ba_7_Nb_4_MoO_20_. *Chem. Mater.* *11*(2), 433-437 (1999).
2. Fop, S. *et al.* High oxide ion and proton conductivity in a disordered hexagonal perovskite. *Nat. Mater.*, **19**, 752-757 (2020).
3. Choi, J.-G., Thompson, L.T.. XPS study of as-prepared and reduced molybdenum oxides. *Applied Surface Science* 93, 143-149 (1996).
4. Kuepper, K., Balasz, I., Hesse, H., Winiarski, A., Prince, K. C., Matteucci, M., Wett, D., Szargan, R., Burzo, E., Neumann, M. Electronic and magnetic properties of highly ordered Sr_2_FeMoO_6_. *Physica Status Solidi* (a), 201, 3252-3256 (2004).
5. Fleisch, T. H., Mains, G. J. An XPS study of the UV reduction and photochromism of MoO_3_ and WO_3_. *The* *Journal of Chemical Physics*, **76**, 780-786 (1982).
6. Ozer, N., Rubin, M. D., Lampert, C. M. Optical and electrochemical characteristics of niobium oxide films prepared by sol-gel process and magnetron sputtering. A comparison.. *Solar Energy Materials and Solar Cells* **40**, 285-296 (1996).
7. Morris, D., Dou, Y., Rebane, J., Mitchell, C. E. J., Egdell, R. G., Law, D. S. L., et al. Photoemission and STM study of the electronic structure of Nb-doped TiO_2_. *Physical Review* B, 61, 13445 (2000).
8. Courths, R., Höchst, H., Steiner, P., Hüfner, S. X-ray and UV photoemission from defects in LiNbO_3_ and BaTiO_3_ surfaces. *Ferroelectrics*, 26, 745-748 (1980).
9. Du, Y.; Kim, D. J.; Varga, T.; Wang, Z.; Szanyi, J.; Lyubinetsky, I. Formation of Single-Phase BaO Nanoclusters. *Thin Solid Films*, **519** (16), 5335–5338 (2011).
10. Tsami, A.; Grillo, F.; Bowker, M.; Nix, R. M. Model NSR Catalysts: Fabrication and Reactivity of Barium Oxide Layers on Cu(1 1 1). *Surf. Sci.*, **600** (17), 3403–3418 (2006).
11. Yashima, M., Tsujiguchi, T., Fujii, K., Niwa, E., Nishioka, S., Hester, J. R., & Maeda, K. (2019). Direct evidence for two-dimensional oxide-ion diffusion in the hexagonal perovskite-related oxide Ba_3_MoNbO_8.5−_ *_δ_*. *J. Mater. Chem. A* **7**, 13910-13916 (2019).
12. Kuang, X., Allix, M., Ibberson, R.M., Claridge, J.B., Niu, H., Rosseinsky, M.J. Oxygen vacancy ordering phenomena in the mixed-conducting hexagonal perovskite Ba_7_Y_2_Mn_3_Ti_2_O_20_. *Chem. Mater.* **19**, 2884-2893 (2007).
13. Gagné, O. C., & Hawthorne, F. C. Comprehensive derivation of bond-valence parameters for ion pairs involving oxygen. *Acta Crystallographica Section B: Structural Science, Crystal Engineering and Materials*, *71*(5), 562-578 (2015).
14. Chen, H., Wong, L. L. & Adams, S. SoftBV – a software tool for screening the materials genome of inorganic fast ion conductors. *Acta Crystallogr. Sect. B Struct. Sci. Cryst. Eng. Mater.* **75**, 18–33 (2019).
15. Fop, S.; Skakle, J. M. S.; McLaughlin, A. C.; Connor, P. A.; Irvine, J. T. S.; Smith, R. I.; Wildman, E. J. Oxide Ion Conductivity in the Hexagonal Perovskite Derivative Ba_3_MoNbO_8.5_. *J. Am. Chem. Soc.* **138**, 16764–16769 (2016).
16. Auckett, J. E., Milton, K. L., Evans, I. R. Cation Distributions and Anion Disorder in Ba_3_*M*NbO_8.5_ (*M* = Mo, W) Materials: Implications for Oxide Ion Conductivity. *Chem. Mater.*, **31**, 1715-1719 (2019).
17. Norby, T. A Kröger-Vink Compatible Notation for Defects in Inherently Defective Sublattices. *J. Korean Ceram. Soc.* **47**, 19–25 (2010).
18. Quarez, E., Noirault, S., Caldes, M. T. & Joubert, O. Water incorporation and proton conductivity in titanium substituted barium indate. *J. Power Sources* **195**, 1136–1141 (2010).
